# On Cupping in the Cure of Hydrophobia

**Published:** 1805-03-01

**Authors:** H. Roxburgensis


### To the Editors of the Medical and Physical Journal.

GENTLEMEN,

IT is always with concern, that we observe the pages of your useful Journal, in place of being occupied with facts, or observations tending to the improvement or the more general diffusion of science, filled with a detail of practices perfectly familiar to every tyro in the school of medicine. We would not be thought by this to discourage unassuming merit, or to insinuate that practitioners ought never to publish the result of their professional experience respecting the virtues of any medicine, or the success of any particular method of treatment, although in very general use; our intention is merely to reprobate the custom of obtruding on the public, as the result of much reflection, and with all the pomp of discovery, opinions and modes of practice, which are to be found in every popular treatise on domestic medicine.

These reflections were suggested to our mind, by observing in the Medical and Physical Journal, for October, the adoption of cupping-glasses proposed as a means of eliminating the poison from wounds inflicted by the bites of rabid animals. In early times, suction, as it is erroneously termed, either by the mouth, or by means of appropriate instruments, was much employed, not only for the removal of every kind of poison in wounds, but also the matter in abscesses, and for various other purposes. Modern practitioners have also availed themselves of the same means to answer similar ends; nor has it been unusual with them, as Mr. Hardman would seem to insinuate, (Medical Journal, page 328) to have recourse to the application of cupping glasses after the use of leeches, when the situation of the parts readily admits of it, and when it is necessary to abstract a greater quantity of blood than would otherwise flow from the wounds made by those animals.

On the subject of hydrophobia, even that sagacious physician Celsus, above a thousand years ago, did not rely on suction alone, in such cases; he at the same time recommends the employment either of the actual cautery, or escharotics. Utique autem, observes that author, in describing the treatment necessary to be pursued against the bites of rabid animals, si rabiosus canis fuit, cucurbitula virus virus ejus extrahendum est. Deinde, si locus neque nervosus, neque musculosus est, vulnus id adurendum est. Si uri non potest, sanguinem homini mitti non alienum est. Tum usto quidem vulneri superimponenda, quæ cæteris ustis sunt. Ei vero, quod expertum ignem non est, ea medicamenta, quæ vehementer exedunt. And again, when treating of the bites of vipers, In primis supra vulnus id membrum deligandum est: non tamen nimium vehementer, ne torpeat. Dein venenum extrahendum est. Id cucurbitula optime facit. Neque alienum est, ante scalpello circa vulnus incidere, quo plus vitiati jam sanguinis extrahatur. Si cucurbitula non est, (quod tamen vix incidere potest) tum quodlibet simile vas, quod idem possit. Si ne id quidem est, homo adhibendus est, qui vulnus exsugat. Galen and Hildanus, as well as many other ancient physicians, likewise recommend the same practice; and were it necessary, we might multiply quotations from every modern author who has written on this subject.

Without entering into the pathological question, whether hydrophobia depends on nervous irritation, or on absorption, or detailing the different arguments by which each of these opinions is supported, we shall, at present, only briefly observe, that as we are unacquainted with any mode of practice, on the success of which, after the accession of the complaint, we can certainly depend, so it must be evident that the most important object for consideration is the means of prevention to be employed, after the bite or application of the virus. To accomplish this end, various measures have been employed; but it may be here not improper to caution the younger members of the profession, against implicitly relying on any mode whatever, unless on the removal of the bitten part, either by excision, or, if that be inadmissible, by means of the actual or potential cautery; after which, cupping, ablution, and other auxiliary means may be resorted to with propriety, as affording additional security. We are far, however, from condemning, in every case, the employment of cupping, and other practices, even in the first instance; for as it may sometimes happen, that the injured part is unfavourable for extirpation, or that the patient will not submit to the operation, in such cases, circumstances must decide the preference of the practitioner; but wherever excision can be accomplished, it ought unquestionably to be adopted, as the most certain means of preventing the occurrence of this dreadful malady.

Since writing the above, we have read the few desultory remarks on Mr. Hume's paper, in the last number of your Journal. Although coinciding in opinion with Mr. Lowe, respecting the inefficiency of cupping as a preventive of hydrophobia, and partaking in some measure of his fears, as to the effect which may be produced on the mind of the young and inexperienced practitioner, by the unqualified recommendation of a practice not wholly to be relied on, we yet conceive it but justice to remark, that in these strictures, the writer appears either to have misunderstood Mr. Hume's meaning, or to have taken an undue advantage of the inaccuracy of his phraseology, in supposing that he intended to recommend cupping as a cure after the accession of the hydrophobic symptoms, since it must, we think, appear sufficiently evident, from the whole scope of the paper in question, that he regards it merely as a prophylactic measure.

In justly reprehending the unqualified praise bestowed by Mr. Hume on the employment of cupping, Mr. Lowe himself seems to have fallen into somewhat a similar error. That caustics, when extensively and properly applied, as well as the excision of the injured part, will prevent the occurrence of the disease, cannot be doubted ; but this is saying nothing more than that if the virus be destroyed the infection will not take place. As, however, in the history of this disease, caustics have been known to fail in the hands of the most expert surgeons, so it is always dangerous, in a dreadful malady like that under consideration, exclusively to direct the attention of practitioners to any one prophylactic mean, when every auxiliary in our power should be resorted to, in the smallest degree calculated to afford additional safety to the unfortunate patient. The solitary case related by Mr. Lowe, in support of the security afforded by the application of caustics against hydrophobia, furnishes no decisive testimony in favour of that practice ; for even admitting that, in this case, the madness of the dog had been unequivocally ascertained, it does not follow as a certain consequence that hydrophobia would have occurred, had no caustic been applied, since it is well known, that, on an average, not above one patient in twenty, or even upwards, injured by an animal actually rabid, become affected by the disease.

H. *Roxburgensis.*

